# Predictors of physical fitness and health-related quality of life based on anthropometrics characteristics and exercise stress test performance in cardiac rehabilitation

**DOI:** 10.1080/07853890.2021.1896644

**Published:** 2021-09-28

**Authors:** Ângela Maria Pereira, Melanie Lameiras, Filipe Matos, Cláudia Torres, Sara Lorga, Luísa Bento

**Affiliations:** a Department of Physical and Rehabilitation Medicine, Hospital Garcia de Orta, Almada, Portugal; b Department of Physiotherapy, Escola Superior de Saúde Egas Moniz (ESSEM), Egas Moniz Cooperativa de Ensino Superior, Caparica, Portugal; c Centro de Investigação Interdisciplinar Egas Moniz (CiiEM), Egas Moniz Cooperativa de Ensino Superior, Caparica, Portugal; d Department of Cardiology, Hospital Garcia de Orta, Almada, Portugal

## Abstract

**Introduction:**

Sedentary lifestyle and physical inactivity are among the leading modifiable risk factors worldwide for cardiovascular disease and all-cause mortality [1]. Many patients in contemporary cardiac rehabilitation programs are quite deconditioned on entry. Cardiac rehabilitation Program (CRP) provides a cost-effective therapy that aims to accelerate recovery following an acute event and reduce the risk of recurrent events, through structured exercise prescription, education, and risk factor modification [2]. The positive effect of CRP on functional capacity has been known for some years [3]. In this study, we aim to assess the relationship between health-related quality of life (HRQoL), Metabolic Equivalents (METS) spend on exercise stress test, body mass index and waist circumference in patients with cardiovascular disease before beginning a cardiac rehabilitation program.

**Material and methods:**

We performed an observational study with inclusion of thirteen male patients with coronary heart disease, 53.8 ± 8.2 years old who were admitted to the Department of Cardiology of Hospital Garcia de Orta and referred for CRP. The HRQoL was assessed with the short form‐36 (SF‐36) questionnaire. METS were calculated using the Bruce protocol when patients performed exercise stress test, body mass index (BMI) and waist circumference was measured, at initial physical examination. All subjects signed an informed consent. This study followed all the principles of Helsinki Declaration.

**Results:**

The mean value for BMI was 28.3 ± 4.0 kg m^−2^, waist circumference 102.6 ± 14.8 cm and METS 9.99 ± 3.14. There was a positive correlation between BMI and waist circumference (*r* = 0.87; *p* = .001); between waist circumference and METS a negative correlation was observed (*r* = −0.59; *p* = .040). The domain physical functioning score from SF-36 was positively correlated with METS (*r* = 0.65; *p* = .010), and was negatively correlated with waist circumference (r = −0.60; *p* = .020). The lowest mean values of SF-36 scores observed were vitality (63.1 ± 20.4), and general health (56.8 ± 22.5).

**Discussion and conclusion:**

As expected subjects with higher BMI had also higher waist circumference. Patients with better perception of physical function were those with lower waist circumference and who had better performance in exercise stress test. These results are in accordance with previous studies [4]. We can conclude that waist circumferences and METS could be good CRP effectiveness predictors on these patients. Once it is expectable to achieve decreases in waist circumferences and increase METS after CRP, with improvements in physical function perception.

## References

[1] Lavie Carl J, Ozemek C, Carbone S, et al. Sedentary behavior, exercise, and cardiovascular health. Circ Res. 2019;124(5):799–815. 30817262 10.1161/CIRCRESAHA.118.312669

[2] Giuliano C, Parmenter BJ, Baker MK, et al. Cardiac rehabilitation for patients with coronary artery disease: a practical guide to enhance patient outcomes through continuity of care. Clin Med Insights Cardiol. 2017;11:1179546817710028. 28638244 10.1177/1179546817710028 PMC5470863

[3] S Listerman J, Bittner V, Sanderson BK, et al. Cardiac rehabilitation outcomes: impact of comorbidities and age. J Cardiopulm Rehabil Prev. 2011;31(6):342–348. 21946420 10.1097/HCR.0b013e31822f189c PMC3219817

[4] Abu-Haniyeh A, Shah NP, Wu Y, et al. Predictors of cardiorespiratory fitness improvement in phase II cardiac rehabilitation. Clin Cardiol. 2018;41(12):1563–1569. 30350419 10.1002/clc.23101 PMC6489768

